# Cytoplasmic HAX1 Is an Independent Risk Factor for Breast Cancer Metastasis

**DOI:** 10.1155/2019/6375025

**Published:** 2019-04-10

**Authors:** Alicja Trebinska-Stryjewska, Lukasz Szafron, Alina Rembiszewska, Maciej Wakula, Sylwia Tabor, Renata Sienkiewicz, Joanna Owczarek, Anna Balcerak, Anna Felisiak-Golabek, Ewa A. Grzybowska

**Affiliations:** Maria Sklodowska-Curie Memorial Cancer Center and Institute of Oncology, Roentgena 5, 02-781 Warsaw, Poland

## Abstract

HAX1 is an antiapoptotic factor involved in the regulation of cell migration and calcium homeostasis, overexpressed in several cancers, including breast cancer. It has been suggested that HAX1 is also implicated in metastasis. Herein we report the results of meta-analysis of* HAX1* expression, based on publicly available data, which confirms its significant overexpression in breast cancer and demonstrates copy number gain and prognostic value of* HAX1* overexpression for metastatic relapse in ER+ tumors. IHC analysis reported here also reveals its significant overexpression in breast cancer samples from primary tumors, indicating significantly higher HAX1 protein levels in a group of patients who developed distant metastases in a disease course. Moreover, we demonstrate that HAX1 localization is important for the prediction of metastatic relapse and that cytoplasmic but not nuclear HAX1 is an independent risk factor for breast cancer metastasis.

## 1. Introduction

Breast cancer is the most common neoplasm and the primary cause of cancer death in women [1]. Breast cancer mortality is almost exclusively due to metastatic disease [2, 3]. The current 5-year survival for primary breast cancer is quite high (80-92%), but, despite the advances in diagnosis and treatment of early breast cancer patients, about 20-40% experience distant organ metastases, for which the prognosis is significantly worse [4–7]. Breast cancer is heterogeneous disease and a probability to develop metastases depends not only on histopathological parameters (lymph node status, histologic grade, and tumor size) but also on molecular subtypes defined roughly as basal-like, normal-like, HER2−enriched, and luminal A and luminal B, each of which has a different prognosis and a pattern of recurrence. For luminal cancers (estrogen and/or progesterone receptor positive) the prognosis is better due to a very effective adjuvant endocrine therapy and the fact that the metastases appear late, often many years after initial diagnosis. Disseminated tumor cells could stay dormant for as long as 20 years but eventually may start to proliferate. Late recurrences were observed in as much as 50% of these cancers [4]. Basal cancers tend to metastasize early (with a peak about 2-3 years after diagnosis) and frequently [8, 9], but typically there is no recurrence after 5 years in this subtype. Distinct pattern of metastatic relapse in basal and luminal subtypes suggests different routes for metastasis.

Better molecular characteristic of the primary tumor is crucial for a good prediction of the clinical outcome. Genetic tests such as MammaPrint (for luminal and basal cancers) [10] and Oncotype DX (luminal cancers only) [11] were developed as a diagnostic tool to predict risk of breast cancer metastasis, based on mRNA expression signature of selected gene sets (70 and 21 genes, resp.). Quantification of the risk of recurrence is especially important for selecting a subset of luminal patients who may benefit from additional chemotherapy and sparing those who would not.

Two other factors have both prognostic and predictive values in breast cancer and are commonly used in risk-assessment: urokinase plasminogen activator protein (uPA) and its inhibitor (PAI-1). ELISA-based assay, developed to assess the levels of both proteins in breast cancer tissues, allows to stratify the patients with node-negative disease into a low-risk group, with a good prognosis without adjuvant chemotherapy and a high-risk group, with high expression of both markers, who may benefit from chemotherapy [12, 13].

Additionally, to monitor a response to the treatment and to assess the probability of metastasis, several blood-based biomarkers have been developed, including Human Epidermal Growth Factor Receptor 2 (HER2), Cancer Antigen 15-3 (CA 15-3, MUC1), and Carcinoembryonic Antigen (CEA) [14].

Herein we assessed the potential of HAX1 expression level in primary tumor samples as an independent prognostic factor for breast cancer metastasis. HAX1 was first characterized in 1997 as an antiapoptotic factor [15] and several reports confirm its involvement in the regulation of apoptosis [16–18]. Additionally, HAX1 was implicated in the regulation of cell motility [19–21] and calcium homeostasis [22].

HAX1 overexpression was reported in several cancers [23–25], including breast cancer [26, 27], and its role in metastasis was suggested in some reports [20, 28]. Sheng and Ni [28] reported that higher HAX1 expression was related to a lower 10-year survival rate in breast cancer patients.

In this report we present data analysis which confirms significant HAX1 overexpression in breast cancer samples, coinciding with high amplification of the* HAX1* gene. Moreover, the analyses reveal significant difference between HAX1 levels in primary tumor samples between nonmetastatic and metastatic groups of patients, indicating that HAX1 may represent an independent risk factor for breast cancer metastasis. Additionally, it was demonstrated that IHC assay which takes into account protein localization may predict clinical outcome more precisely and with a higher strength than mRNA-based estimations.

## 2. Materials and Methods

### 2.1. Study Group

Formalin-fixed, paraffin-embedded (FFPE) tissue samples were collected from breast cancer patients receiving surgical intervention at the Maria Sklodowska-Curie Institute, Oncology Center, between January 2007 and May 2007, after informed consent. The study was approved by Ethics Committee from the Maria Sklodowska-Curie Institute, Oncology Center, Warsaw, in accordance with the guidelines of the Helsinki Declaration of 1975, revised in 1983. Clinical data and histology reports for each patient were reviewed by two clinicians and a pathologist, respectively. De-identified patients data were accessed using MedStream Designer platform (Transition Technologies S.A.). Patients were retrospectively analyzed and divided into two groups: with and without distant metastases within the follow-up period of 9 years. Inclusion criteria for the study were as follows: invasive breast cancer stages I-III, the absence of distant metastasis at the time of surgery, and the presence of tumor tissue in FFPE confirmed by a pathologist. Exclusion criteria were as follows: previous history of breast cancer, previous or simultaneous history of any other malignances, and neoadjuvant chemotherapy. A total of 46 patients who met the inclusion and exclusion criteria were selected: 23 consecutive patients with distant metastases and 23 consecutive patients without distant metastases within the follow-up period (comparative group). Assuming a power of 80%, *α*=0.05, and normal distribution of data, a number of patients tested would allow to detect a difference of at least 0.43 between group means (with a common standard deviation equal to 0.5) when analyzed with Mann-Whitney U test.

Patients' age at the time of diagnosis, estrogen receptor (ER) status, progesterone receptor (PGR) status, HER status, tumor size (pTNM scale), node status (pTNM scale), clinical stage (AJCC Anatomic Stage Group), histological grade (Nottingham Histologic Score system), histology, and molecular subtype (based on routine immunohistochemical evaluation of ER, PGR, HER2 and Ki-67) were recorded. Patients' characteristics are shown in Table S1.

### 2.2. Follow-Up and Outcomes

Patients' records were tracked from the time of surgery until May 2016. Information about clinical outcomes (distant metastases confirmed by imaging or histologic evidence, death from any cause) was retrieved from clinical records and The National Cancer Registry in Poland. Distant relapse-free survival (DRFS) was defined according to STEEP system [34] as the time from surgical intervention until the time of distant recurrence, death from any cause, or the last follow-up. Complete events in DRFS analysis were distant metastasis or death, whichever came first. Overall survival (OS) was defined as the time from surgery to the last follow-up (censored event) or to the time of death from any cause (complete event).

### 2.3. Immunohistochemistry

Immunohistochemical staining with a monoclonal mouse anti-HAX1 antibody (BD Biosciences) or a control mouse IgG of the same subclass was performed as described previously [27] on a set of representative slides from formalin-fixed, paraffin-embedded breast tumors. HAX-1 expression was scored manually according to Ball et al. [35]. It was evaluated independently for nuclear and cytoplasmic staining. Light microscopy evaluation at 400x magnification was used to count 100 tumor cells within areas of the strongest staining. Each nucleus and cytoplasm in a given field was assigned to an intensity category of 0 (absent), 1 (weak), 2 (moderate), or 3 (strong). The percentage of cells in each intensity category was determined as N0, N1, N2, and N3, respectively. A distribution score (ID score) was then calculated as

(1) $$\begin{array}{c} ID=\frac{\left(/N0*0/+/N1*1/+/N2*2/+/N3*3/\right)}{100}. \end{array}$$

The ID score therefore ranged from 0 (absent staining in all cells) to a maximum 3 (100% cells having a staining intensity of 3). The values of total HAX1 staining were obtained by adding nuclear and cytoplasmic ID scores for each sample.

### 2.4. Immunofluorescence

MCF7 (ATCC), MDA-MB-231 (ATCC), and HeLa (ATCC) human cell lines were used in the experiments. All cell lines were authenticated by Eurofins Genomics (Germany). Cells were grown in Dulbecco's Modified Eagle Medium supplemented with 10% fetal bovine serum (Thermo Fisher Scientific). Immunofluorescence was performed as described previously [36] with primary anti-HAX1 antibody (rabbit, 1:100, Thermo Fisher Scientific) and secondary goat anti-rabbit Alexa Fluor 594 antibody (1:500, Thermo Fisher Scientific). Cells were observed using the Zeiss LSM 800 confocal microscope. Images represent single Z-stacks. Colocalization was quantified using ImageJ JACoP plugin [37], for 3-5 independent fields of vision and approximately 25-60 cells per field.

### 2.5. Database Gene Expression Analysis

*HAX1 *gene expression in primary breast cancer compared to normal breast tissue was analyzed using the Oncomine™ Platform (Thermo Fisher, Ann Arbor, MI) [38, 39].* HAX1* expression (RNAseq) in breast cancer in relation to phenotypic variables was explored in TCGA-BRCA cohort (data generated by the TCGA Research Network: http://cancergenome.nih.gov/) using Xena Functional Genomics Explorer (https://xenabrowser.net/, 2018) and in a set of microarray data using bcGenExMiner (http://bcgenex.centregauducheau.fr, 2018) [33].

### 2.6. Database Copy Number Variation and Mutation Analysis

Genomic alterations (mutations, gene amplification, and/or deletion) of* HAX1* gene in breast cancer were assessed using cBioPortal for Cancer Genomics (http://www.cbioportal.org/index.do, 2018) [40, 41]. The following cohorts of invasive breast carcinoma were included: Breast Cancer (METABRIC) [29, 42], Breast Invasive Carcinoma (British Columbia) [43], Breast Invasive Carcinoma (Broad) [44], Breast Invasive Carcinoma (Sanger) [45], Breast Invasive Carcinoma (TCGA, PanCancer Atlas) [30], Mutational profiles of metastatic breast cancer (France) [46], and the Metastatic Breast Cancer Project (Provisional, April 2018). The other cohorts were excluded from the analysis due to patients overlapping or the difference in sample type (xenografts instead of primary tumor). Groups with shallow deletion (possibly heterozygous deletion), diploid status, gain, or high-level amplification of* HAX1* gene generated by GISTIC algorithm [47] were compared for mRNA expression in METABRIC and TCGA cohorts. For the latter,* HAX1* mRNA levels were also correlated with log2 copy number values using Pearson's correlation coefficient.

*HAX1* copy number variation in primary breast cancer in comparison to normal tissues was also analyzed on the Oncomine Platform using the following threshold values: p-value 0.05, fold change ‘all', and gene rank ‘top 5%.'

### 2.7. Database Survival and Prognostic Analysis

Survival analyses of patients stratified according to* HAX1 *expression were performed using KM Plotter (http://kmplot.com/analysis/, 2018) [48]. HAX1 expression levels based on Affymetrix probe ID 201145_at in 35 cohorts of breast cancer patients deposited in GEO database (Gene Expression Omnibus, NCBI) were used. The general settings were as follows: patients split by median or by best cut-off; follow-up threshold: all; probe set options: only JetSet best probe set; quality control: removing redundant samples and excluding biased arrays.

Survival analysis of patients with metastatic relapse information was also performed using Breast Cancer Gene-Expression Miner v4.1 (bcGenExMiner v4.1) [33]. Patients were split into two groups according to gene's expression median and Kaplan-Meier survival curves were plotted for each group. Breast Cancer Gene-Expression Miner v4.1 was also used in targeted prognostic analysis of* HAX1* gene expression for all patients with metastatic relapse information. The results summarize univariate Cox scores (hazard ratios, p-values) for each cohort fulfilling the chosen criteria and all of these cohorts pooled together. The results were presented in forest plot. Additionally, multivariate Cox scores (adjusted on NPI/AOL) were calculated for* HAX1*.

### 2.8. Statistical Analysis

Statistical analysis was performed using SAS Enterprise Guide 7.11 (Copyright ©2015 by SAS Institute Inc., Cary, NC, USA) and Stata software (StataCorp., College Station, TX, StataCorp LP.). GraphPad Prism version 6.07 for Windows (GraphPad Software, La Jolla, California USA, www.graphpad.com) was used to visualize data. O'Brien-Castelloe approximation (SAS Enterprise Guide 7.11) was employed for statistical power and sample size analysis for Mann-Whitney U test. Baseline demographics, tumor characteristics, and types of treatment were compared between the group of patients with distant metastases and the comparative group using the Mann-Whitney U test for continuous and ordinal variables and by Pearson's chi-squared test for categorical variables. The Shapiro-Wilk W test was used to determine whether HAX1 protein levels measured by immunohistochemistry were normally distributed. The associations between HAX1 immunoreactivity and progression, along with clinicopathological parameters, ER, PGR, and HER2 status, were assessed using the Mann-Whitney U test or Kruskal-Wallis test, depending on whether the nominal variable had two or more categories. If significant, the Kruskal-Wallis test was followed by pairwise comparisons using the Mann-Whitney U test. Receiver operating characteristic (ROC) curve analysis was performed to determine the overall test performance and to calculate possible cutoff points for HAX1 protein levels. Optimal cutoff values were calculated using the nearest to (0,1) method and the maximum value of the Youden index. Kaplan-Meier survival analyses were carried out for overall survival (OS) and distant relapse-free survival (DRFS). The log-rank test was used to evaluate the equality of survivor function for groups with lower and higher HAX1 expression categorized according to values obtained in the ROC curve analysis. The Cox proportional hazards model was used for univariate and multivariate analyses of patient survival depending on HAX1 expression, categorized as described above. In the multivariate survival analyses, HAX1 levels were assessed along with the following variables: PGR expression (categorization: positive vs. negative), clinical stage (I vs. II vs. III), histological grade (1 vs. 2 vs. 3), and molecular subtype (luminal vs. others). Hazard ratios (HR) with 95% confidence intervals and p-values were reported (Table 1).* HAX1* gene expression retrieved from databases was compared in different subgroups using Student's t-test and one-way Welch's or Fisher's ANOVA followed by post-hoc Tukey-Kramer test. All tests used in this study were two-tailed and the significance level (alpha) was always set to 0.05.

## 3. Results

### 3.1. HAX1 Is Significantly Overexpressed in the Majority of Analyzed Datasets of Breast Cancer Primary Tumor Samples

**Figure 1:**
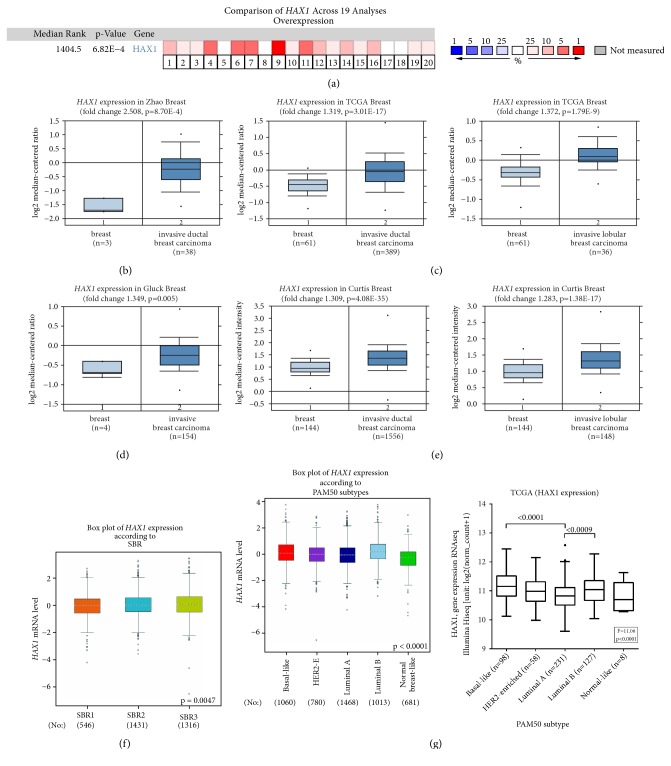
HAX1 is overexpressed in primary breast tumor in comparison to normal breast tissue. (a-e)* HAX1 *expression in invasive breast cancer (ductal and lobular) in comparison to normal breast tissue assessed in publicly available datasets on the Oncomine Platform. (a) Comparison of* HAX1* overexpression across 19 analyses. Dataset legend in Figure S1. (b-e)* HAX1* overexpression in selected datasets [29–32]. Differences between groups were assessed by Student's t-test and results with p-values <0.05 were considered significant. (f)* HAX1 *expression in breast cancer samples stratified according to grade (Scarff-Bloom-Richardson grade, SBR) analyzed using bcGenExMiner. (g)* HAX1 *expression in breast cancer samples stratified according to molecular subtype (PAM50 classification) in a set of microarray data analyzed using bcGenExMiner [33] (left panel) or RNAseq TCGA-BRCA data (right panel). Differences between groups in (f) and (g) were assessed by Welch's or Fisher's ANOVA followed by post-hoc Tukey-Kramer test.

*HAX1 *overexpression in primary breast cancer in comparison to normal breast tissues was identified at mRNA [26, 27] and protein level [27]. To further confirm this observation we performed analysis on breast cancer cohorts using the Oncomine Platform [38, 39] and taking into account invasive breast cancer samples (ductal and lobular).* HAX1 *gene expression in invasive primary tumor was significantly elevated compared to normal tissue in 16 out of 19 analyses (Figure 1(a), legend in Figure S1). Detailed analyses for ductal and lobular carcinoma in selected datasets confirmed these conclusions (Figures 1(b)–1(e)).

*HAX1* expression in breast cancer in relation to phenotypic variables was assessed in a set of microarray data using Breast Cancer Gene-Expression Miner v4.1 (bcGenExMiner) [33]. This analysis revealed that* HAX1* expression correlates positively with grade (Figure 1(f)), confirming our previous results, obtained on a small group of patients [27]. It was also observed that* HAX1* expression differs significantly in molecular subtypes of breast cancer, with the highest expression in basal and luminal B subtypes, associated with more aggressive neoplasm (Figure 1(g)).

### 3.2. HAX1 Gene Copy Number Is Altered in Breast Cancer Patients

**Figure 2:**
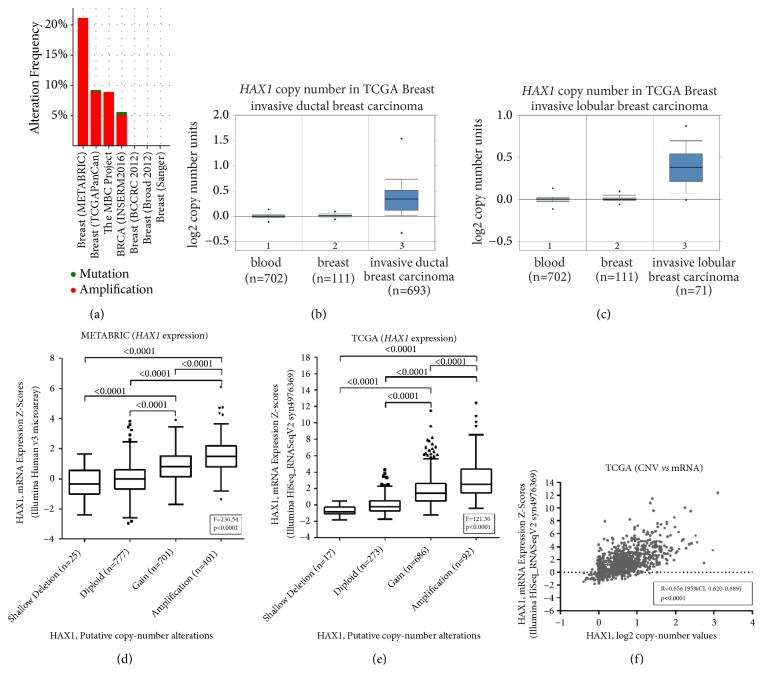
*HAX1* gene copy number is altered in breast cancer patients. (a) Alterations in* HAX1 *gene analyzed in 7 cohorts of invasive breast carcinoma patients using cBioPortal for Cancer Genomics. (b-c)* HAX1* gene copy number in TCGA-BRCA data from the Oncomine Platform for (b) invasive ductal carcinoma and (c) invasive lobular carcinoma compared to blood and normal breast tissue. (d-e) Comparison of* HAX1* expression in primary breast cancer samples in relation to DNA copy number in (d) METABRIC cohort [29] and (e) TCGA-BRCA cohort [30]. Differences between groups were assessed by Fisher's ANOVA followed by post-hoc Tukey-Kramer test. (f) Correlation of* HAX1 *expression and log2 copy number values in TCGA-BRCA cohort (Pearson's correlation coefficient).

Analysis of* HAX1* gene in 7 cohorts of invasive breast carcinoma patients using cBioPortal for Cancer Genomics [40, 41] revealed* HAX1* altered status in 15% (549/3655) of sequenced cases. Only three patients had mutations in* HAX1* sequence, one truncating E59X and two missense mutations, E39K and P259A. Majority of the identified alterations comprised of high-level gene amplification which was detected in all 4 cohorts containing copy number variation data [29, 30, 42, 46] and ranged from 5.16% to 21.06% of all cases (average 16.01%) (Figure 2(a)). Additionally, low-level* HAX1* gene gain was identified in 36.81% to 63.86% cases (average 45.92%) whereas shallow deletion (possibly heterozygous deletion) was present in only 0% to 3.76% of patients (average 1.49%). Additionally, log-2* HAX1 *gene copy number units were compared between blood, breast, and invasive ductal and invasive lobular carcinoma in TCGA-BRCA cohort using the Oncomine Platform and were found to be elevated for both invasive ductal carcinoma (fold change: 1.273, p=9.26E-135, gene rank: top 1%, Figure 2(b)) and invasive lobular carcinoma (fold change: 1.297, p=1.13E-22, gene rank: top 2%, Figure 2(c)).

Two cohorts analyzed using cBioPortal, METABRIC [29], and TCGA-BRCA [30] contained gene expression information so it was possible to relate* HAX1* gene copy number with* HAX1 *mRNA level. In both cohorts mRNA expression differed significantly between putative groups with diploid DNA content and* HAX1 *gene gain or amplification (Figures 2(d) and 2(e)). In TCGA cohort HAX1 log2 copy number values showed a moderate positive correlation with mRNA expression (Pearson's r=0.656, p<0.0001, Figure 2(f)).

### 3.3. HAX1 Overexpression Is Associated with Cancer Relapse and Has Prognostic Impact on ER+ Subset

**Figure 3:**
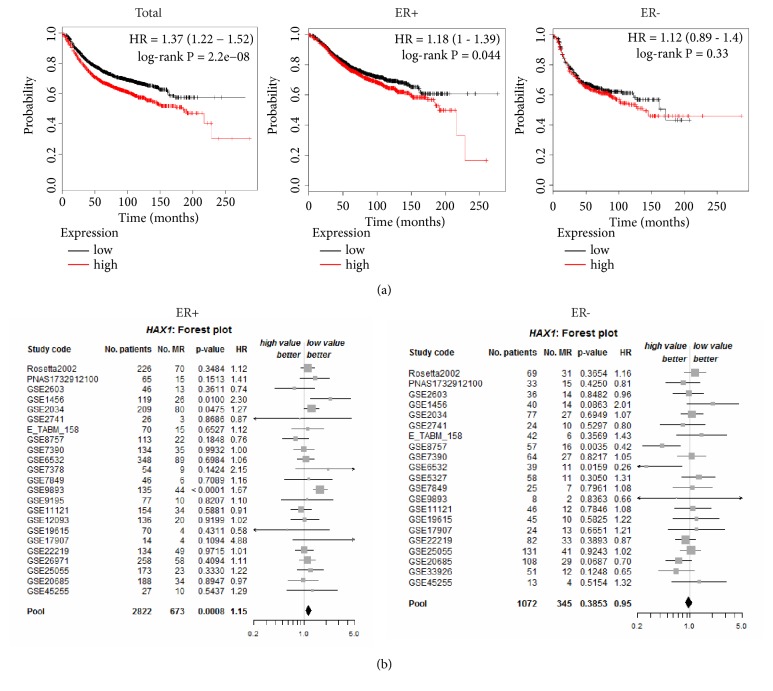
*HAX1* overexpression is higher in patients with breast cancer relapse and has prognostic impact on ER+ subset of patients. (a) RFS analysis for total number of patients and subsets with different ER status (left: ER+; right: ER-). Patients were split into groups with high and low* HAX1* expression (based on microarray data, split by median). Kaplan-Meier estimates were generated in KM Plotter online tool for all data available for 2017 (merged datasets). Probability of cancer relapse is plotted against time. (b) Forest plots estimating prognostic impact of* HAX1* expression in ER+ (n = 2,822) and ER- (n = 1,072) subsets of patients with metastatic relapse information (bcGenExMiner). Values in columns represent summarized univariate Cox scores (p-values, hazard ratios) for each cohort fulfilling the chosen criteria and for pooled cohorts. MR: metastatic relapse.

Survival analyses of breast cancer patients stratified according to* HAX1 *expression were performed using KM Plotter and microarray data from 35 breast cancer cohorts from GEO (Gene Expression Omnibus, NCBI). RFS (relapse-free survival) analysis including 3,951 patients showed a statistically significant difference in survival, favoring patients with lower* HAX1 *expression regardless of whether patients were split by median (HR=1.37, 95% CI, 1.22-1.52, log-rank p=2.2E-08, Figure 3(a)) or best cutoff value (HR=1.42, 95% CI, 1.27-1.58, log-rank p=3.6E-10, FDR=1%). OS (overall survival) analysis in 1,402 patients also indicated statistically significant more favorable prognosis for patients with lower* HAX1* expression, but only if patients were split by best cutoff value (HR=1.41, 95%CI, 1.12-1.77, log-rank p=0.0034) and at the expense of false discovery rate (FDR=50%) (Figure S2A).

In breast cancer ER status is one of the most important prognostic and predictive factors. Therefore, RFS analysis was performed using KM Plotter on subgroups of breast cancer patients with different ER status, using median value of* HAX1 *expression to avoid high values of FDR. Statistical significance was detected for ER+ (n=2,061) but not ER- (n=801) subgroup of patients (HR=1.18, 95% CI, 1.00-1.39, log-rank p=0.044 and HR=1.12, 95% CI, 0.89-1.40, log-rank p=0.33, resp., Figure 3(a)).

Prognostic analysis was carried out using bcGenExMiner [33]. Targeted prognostic analysis for* HAX1* in a group of ER-positive patients with metastatic relapse information (n = 2,822) revealed statistical significance (HR=1.15, p-value=0.0008) for* HAX1* expression level in the pooled cohort (Figure 3(b), left panel). Additionally, to evaluate independent prognostic impact of* HAX1* in ER+ patients relative to the well-established breast cancer prognostic indexes, including Nottingham Prognostic Index (NPI) [49] and Adjuvant! Online (AOL) [50], adjusted Cox proportional hazards model was used, revealing statistical significance for* HAX1* expression adjusted on AOL (HR 1.27, 95% CI 1.06-1.52, p-value: 0.0108, 12 cohorts, 382 patients, 101 metastases).

The same analysis performed for patients with negative ER status (n=1,072) revealed the lack of statistical significance (p-value=0.3853 for the pooled cohort) and even the tendency for better prognosis associated with HAX1 overexpression (Figure 3(b), right panel).

Prognostic analysis performed for* HAX1* expression regardless of ER status (n=3,924) indicated significance, but bordering on the 0.05 threshold (HR=1.07, 95% CI, 1.00-1.14, p-value: 0.0432, Figure S2B). Additionally, KM curves for metastatic relapse-free survival (MRFS) were plotted in bcGenExMiner for each group of patients with metastatic relapse information (all patients, ER+, ER-), and the results were consistent with the previous RFS analyses, showing significant difference for ER+ group of patients and the lack of significance in ER- group of patients (Figure S2C).

### 3.4. Cytoplasmic HAX1 Levels Are Significantly Higher in the Primary Tumor of Breast Cancer Patients Who Experience Distant Metastasis during the Disease Course

**Figure 4:**
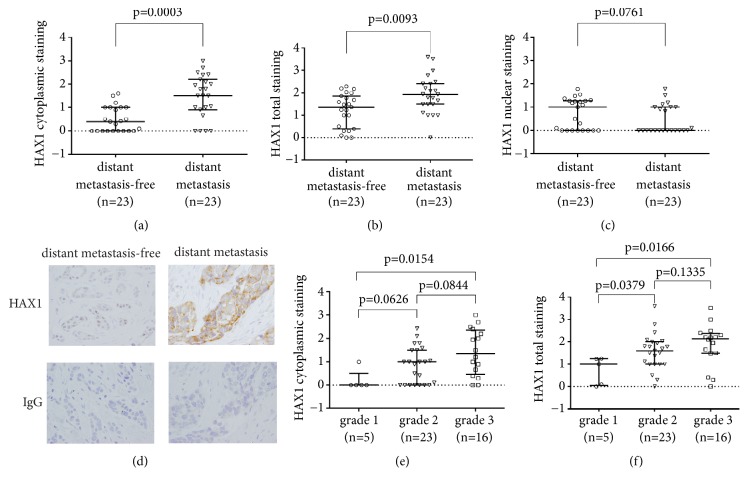
HAX1 protein level in primary tumors stratified according to selected clinical and histological factors (presence of distant metastases, tumor grade). (a-d) HAX1 protein levels in the primary tumor were quantified from IHC data in distant metastasis-free versus distant metastasis group for (a) cytoplasmic, (b) total, and (c) nuclear HAX1 staining. (d) Representative images of HAX1 IHC and negative isotype control for patients from metastasis-free versus distant metastasis group. ×40 objective, bar: 100 *μ*m. (e) Cytoplasmic and (f) total HAX1 staining in breast cancers stratified according to tumor grade (grades 1-3). Results for individual patients and median and interquartile range for each group are shown. Differences in HAX1 protein levels between groups were assessed by the Mann-Whitney U test and results with p-values <0.05 were considered significant.

46 breast cancer patients who were free of distant metastasis at the time of surgery and received no neoadjuvant therapy were retrospectively analyzed for HAX1 protein levels (cytoplasmic and nuclear) in primary tumors by immunohistochemistry. Half of the analyzed group developed distant metastasis during a follow-up period of 9 years. Cytoplasmic HAX1 protein levels were significantly elevated (p=0.0003) in the group of patients with distant metastasis (median 1.50, mean±SD 1.48±0.92, 95% CI of the mean 1.08-1.87) compared to the group with no distant metastasis (median 0.40, mean±SD 0.50±0.54, 95% CI of the mean 0.26-0.73) (Figure 4(a)). Total HAX1 staining was also higher in the distant metastasis patient group, although the effect was less prominent (metastasis: median 1.94, mean±SD 1.93±0.85, 95% CI of the mean 1.56-2.29 vs. metastasis-free: median 1.37, mean±SD 1.22±0.76, 95% CI of the mean 0.89-1.55, p=0.0093) (Figure 4(b)). The opposite effect, albeit not statistically significant, was observed for nuclear HAX1 levels (metastasis: median 0.00, mean±SD 0.45±0.60, 95% CI of the mean 0.19-0.71 vs. metastasis-free: median 1.00, mean±SD 0.73±0.66, 95% CI of the mean 0.44-1.01, p=0.0761) (Figure 4(c)). Representative images of the typical staining in metastasis-free and metastatic groups are presented in Figure 4(d).

The two analyzed groups were well matched, as patients' clinicopathological parameters and treatment did not differ significantly except for PGR status (Table S1). Analyses of HAX1 protein levels in groups stratified according to known prognostic factors showed that the values of the cytoplasmic and total HAX1 signal were positively associated with tumor grade (Figures 4(e) and 4(f), resp.), but not other prognostic factors.

### 3.5. High Cytoplasmic and Total HAX1 Protein Levels in Breast Cancer Cells Are Risk Factors for Distant Metastasis and Death

To ascertain if HAX1 protein levels in primary tumor can be used as a prognostic factor in breast cancer, we analyzed follow-up patient data and recorded time to distant recurrence and/or time to death from any cause for all 46 patients. The total follow-up time was 9 years; 61% of the patients had been followed for a minimum of 5 years. 23 patients developed distant metastasis. 23 out of 46 patients were still alive at the end of the follow-up period (18 in a group with no distant metastasis and 5 in a group with distant metastasis).

**Figure 5:**
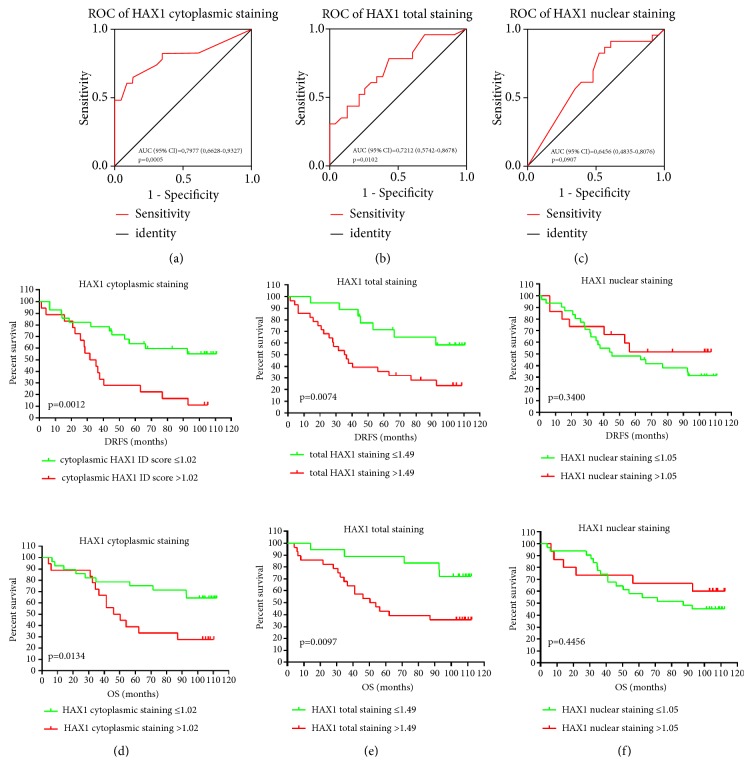
HAX1 protein level in primary tumor is a risk factor for breast cancer progression. (a-c) Receiver operating characteristic analysis for (a) cytoplasmic, (b) total, and (c) nuclear HAX1 protein levels was performed to define the best cutoff values for subsequent survival analysis. Area under curve (AUC) with 95% CI and p-values for each ROC curve are shown. (d-f) Kaplan-Meier survival estimates for distant recurrence-free survival (DRFS) and overall survival (OS) in breast cancer patients according to proposed cutoff values of (d) cytoplasmic HAX1 protein levels ≤1.02 (n=28) versus >1.02 (n=18), (e) total HAX1 protein levels ≤1.49 (n=18) versus >1.49 (n=28), and (f) nuclear HAX1 protein levels ≤1.05 (n=31) versus >1.05 (n=15). The log-rank test was used to evaluate the equality of survivor function for groups with lower and higher HAX1 expression and p-values <0.05 were considered significant.

Receiver operating characteristic (ROC) analysis was performed to define the best cutoff value of the HAX1 signal and to measure the overall test performance which would use HAX1 protein levels to predict breast cancer metastasis. The analysis was done separately for cytoplasmic, total, and nuclear HAX1 immunohistochemical staining (Figures 5(a)–5(c)). The highest value of area under the curve (AUC) was obtained for cytoplasmic HAX1: 0.7977 (95% CI 0.6628-0.9327, p=0.0005) (Figure 5(a)). The best cutoff points for cytoplasmic, nuclear, and total HAX1 were, respectively, 1.02 (sensitivity 0.65 and specificity 0.87), 1.05 (sensitivity 0.82 and specificity 0.48), and 1.49 (sensitivity 0.78 and specificity 0.57).

Cutoff points estimated from ROC curves were used in subsequent survival analyses by the Kaplan-Meier method. The log-rank test showed a significant difference favoring, for both distant recurrence-free survival (DRFS) and overall survival (OS), patients with a cytoplasmic HAX1 ID score of ≤1.02 (p=0.0012 and p=0.0134, resp., Figure 5(d)). 43% of patients in the group with cytoplasmic HAX1 protein levels ≤1.02 experienced distant metastasis/death within 9 years compared to 89% of patients in the group with cytoplasmic HAX1 protein levels >1.02. Overall survival analysis showed that, at the end of follow-up, 64% of patients were still alive in the group with a cytoplasmic HAX1 of ≤1.02 compared to 28% of patients in the group with cytoplasmic HAX1 >1.02. Similar results were observed for total HAX1 protein levels. Patients with total HAX1 protein levels ≤1.49 showed significantly increased DRFS and OS compared to the group with a total HAX1 protein level of >1.49 (p=0.0074 and p=0.0097, resp., Figure 5(e)). Nuclear HAX1 staining showed no prognostic value for neither DRFS nor OS (Figure 5(f)).

Overall survival (OS) and distant recurrence-free survival (DRFS) for 46 breast cancer patients were also evaluated by 3 different univariate and multivariate analyzes, in which the HAX1 protein expression, either cytoplasmic, nuclear, or cumulative, was assessed by IHC (Table 1). We found out that elevated HAX1 levels in the cytoplasm emerged as an independent, negative prognostic factor, associated with an increased risk of distant metastasis (HR 2.832, 95% CI 1.207-6.644, p=0.017). Correspondingly, the results obtained for cumulative expression of HAX1 also showed its adverse effect on DRFS (HR 4.249, 95% CI 1.404-12.86, p=0.010). HAX1 nuclear expression had no impact on survival.

### 3.6. HAX1 Localization Varies among Breast Cancer Cell Lines

**Figure 6:**
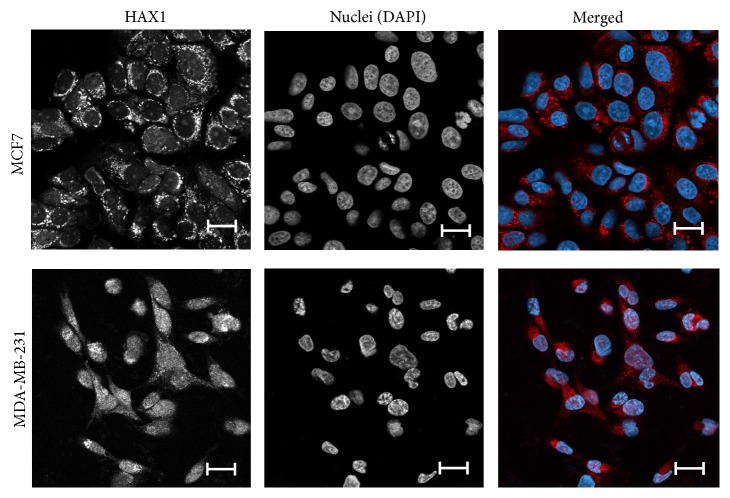
HAX1 localization in breast cancer cell lines of different characteristics. Endogenous staining of HAX1 (red) and nuclei (DAPI, blue) in MCF7, luminal-like epithelial cells and MDA-MB-231, basal-like cells after epithelial-mesenchymal transition. Bar: 20 *μ*m.

Endogenous HAX1 protein was detected by immunofluorescence in luminal-like MCF7 and basal-like MDA-MB-231 cell lines, revealing significant differences. In MCF7 cells HAX1 staining was mostly cytoplasmic, while in MDA-MB-231 HAX1 was also detected in the nuclei (Figure 6). Nuclear colocalization was calculated using ImageJ JACoP, showing a significant shift of Pearson's correlation coefficient (PCC) and two Mander's overlap coefficients (M1, M2) from 0.101 (PCC), 0.207 (M1), and 0.116 (M2) in MCF7 cells to 0.467 (PCC), 0.377 (M1), and 0.592 (M2) in MDA-MB-231 cells, respectively (p-values for PCC, M1, and M2: 0.0105, 0.0328, and 0.0181, resp.).

## 4. Discussion

Advancing on our previous study [27], in which we demonstrated* HAX1* overexpression in breast cancer and its differential localization (cytoplasmic and nuclear), we expanded our analysis to assess its effect on metastasis. Database analysis on large group of patients confirmed* HAX1* overexpression in breast cancer samples, which tallies with the previous study by Luo et al. [26]. The analysis revealed also the correlation of HAX1 overexpression with tumor grade, which is consistent with our previous [27] and current IHC results. Additionally,* HAX1* overexpression was shown to correlate with gene amplification. Although there were several studies reporting* HAX1* overexpression in different types of malignancies, we showed for the first time that high* HAX1 *mRNA levels in cancer cells could be a consequence of gene amplification, at least in breast cancer. Detailed analysis of* HAX1* expression in molecular subtypes demonstrated that the highest overexpression was observed in basal and luminal B subtypes, which are more aggressive.

Database analysis of* HAX1* expression in correlation to metastasis revealed its significant prognostic value for luminal (ER+) subset while for ER-, despite high overexpression in basal cancers, the expression level had no prognostic value. This apparent paradox can be resolved on the basis of cellular localization.

Our previous IHC analysis [27] indicated two different localizations of HAX1 protein in breast cancer tumor samples: cytoplasmic and nuclear. Nuclear localization of HAX1 was also reported in cell lines [36] and rat tests [51]. Different localization may translate into different functionality and different impact on tumor progression, as in case of Aurora A kinase, where nuclear protein acquires kinase-independent transactivating function, which enhances breast cancer stem cell phenotype [52]. Thus, in this report, we have analyzed HAX1 protein levels in the primary tumor of breast cancer patients divided into metastatic and nonmetastatic groups. IHC analysis enabled us to differentiate between cytoplasmic and nuclear localization of HAX1. Overall, our results demonstrated that HAX1 protein level is significantly higher in metastatic group of patients, but this effect can be observed only for evaluations concerning cytoplasmic and total HAX1, while for nuclear localization it does not exist and the trend is even opposite (less HAX1 in metastatic group). Clearly, the results for total HAX1 levels are influenced by the cytoplasmic subset, for which the difference is huge.

Thus, our evaluation of HAX1 protein levels and localization in the samples from metastatic versus nonmetastatic groups of patients indicates a positive relationship between HAX1 cytoplasmic expression and the occurrence of a secondary tumor at distant locations in the course of the disease (opposite to the relations observed for Aurora A). High cytoplasmic HAX1 level is associated negatively with progression-free survival and overall survival. Similar results were obtained for total HAX1; however, ROC curve analysis indicated a higher ability to identify patients at risk of progression when using cytoplasmic, not total HAX1 levels.

Accordingly, the experimental immunofluorescence results showing that HAX1 localization is more cytoplasmic in luminal-like than basal-like cell lines can explain the apparent difference in HAX1 prognostic value for ER+ and ER- subsets. It seems plausible that, in basal cells, despite the high HAX1 expression, nuclear localization of HAX1 prevents its prometastatic action, by assuming different functionality or simply by sequestering cytoplasmic HAX1. Alternatively, it seems plausible that nuclear HAX1 can block and/or sequester in inactive complexes some nuclear factor(s), specific to luminal cancers, whose action is linked to metastasis, probably by the regulation of transcription. Thus, nuclear HAX1 would have protective effect (restricted to luminal-like cells), which would not be present in cells with cytoplasmic HAX1. Of note, HAX1 was shown to bind directly estrogen receptor [53], which is a prometastatic factor. Estrogen responsiveness is one of the main factors differentiating between the two cell lines used in experiments. However, since luminal patients are treated with anti-estrogen therapy, some other factors may be involved. Further research should explain specific molecular mechanisms of function for cytoplasmic and nuclear HAX1, which contribute to metastasis.

## 5. Conclusions

Overall, presented results indicate* HAX1* overexpression, copy number gain, and prognostic impact on metastatic breast cancer. Moreover, cytoplasmic but not nuclear HAX1 demonstrated to be an independent, negative prognostic factor for breast cancer metastasis.

Multigene tests like MammaPrint and Oncotype DX have a proved prognostic value, but they rely on expression on mRNA level, which does not always accurately correspond to protein level and does not consider the factor of protein localization. It is possible that for some markers establishing the level of localized protein provides more accurate prediction than mRNA profiling. Further analysis is required to confirm its prognostic value in the clinic, but assessing HAX1 protein levels and localization in primary tumor samples may become a useful tool for estimating the probability of luminal breast cancer dissemination.

## Figures and Tables

**Table 1:** Multivariate Cox regression analysis of HAX1 levels in human breast cancers.

*Evaluation of HAX1 levels in the cell nuclei*		

*Variable name*	*OS (23/46)* ^*1*^	*DRFS (28/46)* ^*1*^
	HR (95% CI), p	HR (95% CI), p

HAX1 (≤1.05 vs. >1.05)	NS	NS
Histological grade (2 vs. 1)	6.06E+8 (2.26E+8-1.63E+9), <0.001	NS
Clinical stage (III vs. I)	3.171 (1.017-9.884), 0.047	NS

*Evaluation of HAX1 levels in the cytoplasm*		

*Variable name*	*OS (23/46)*	*DRFS (28/46)*
	HR (95% CI), p	HR (95% CI), p

HAX1 (≤1.02 vs. >1.02)	NS	*2.832 (1.207-6.644), 0.017*
Histological grade (2 vs. 1)	4.86E+8 (1.74E+8-1.36E+9), <0.001	NS

*Evaluation of HAX1 levels in both of the nuclei and the cytoplasm*		

*Variable name*	*OS (23/46)*	*DRFS (28/46)*
	HR (95% CI), p	HR (95% CI), p

HAX1 (≤2.06 vs. >2.06)	NS	*4.249 (1.404-12.86), 0.010*
Histological grade (2 vs. 1)	3.44E+8 (1.17E+8-1.01E+9), <0.001	NS

The multivariate analysis of prognosis was carried out using the Cox proportional hazards model. ^1^Values before and after a slash (/) stand for the number of complete observations versus all observations, respectively. Only the results with p-values <0.05 are shown and those with p-values <0.05 for HAX1 expression are highlighted in italic type. HR and CI stand for the hazard ratio and confidence interval, respectively. OS: overall survival; DRFS: distant recurrence-free survival; NS: a nonsignificant result (p ≥0.05).

## Data Availability

The data used to support the findings of this study are available from the corresponding author upon request.

## Supplementary Materials

Supplementary Materials Table S1: clinical and pathological characteristics of breast cancer patients. Figure S1: dataset legend to Oncomine meta-analysis of* HAX1* expression in breast cancer (ductal and lobular). Figure S2:* HAX1* overexpression is associated with metastatic relapse and survival (additional data).
