# Supplementary material for: Cytoplasmic HAX1 Is an Independent Risk Factor for Breast Cancer Metastasis
Source: J Oncol. 2019 Apr 10;2019:6375025. doi: 10.1155/2019/6375025 (PMC6481036; doi:10.1155/2019/6375025)
Supplement: Supplementary Materials — Table S1: clinical and pathological characteristics of breast cancer patients. Figure S1: dataset legend to Oncomine meta-analysis of HAX1 expression in breast cancer (ductal and lobular). Figure S2: HAX1 overexpression is associated with metastatic relapse and survival (additional data). [file 6375025.f1.pdf]

Supplementary materials

Table S1 Clinical and pathological characteristics of breast cancer patients.

	All patients			Comparative group			Distant metastasis group			P-value
	values	no.	%	values	no.	%	values	no.	%	
**Patients**		46	100.0		23	50.0		23	50.0	
**Age at diagnosis (years)**										0.6445[a]
median	59.0			58.6			61.5			
range	22.9-84.4			30.5-84.4			22.9-75.6			
**Menopausal status**										0.7241[b]
pre		8	17.4		5	21.7		3	13.0	
post		29	63.0		14	60.9		15	65.2	
unknown		9	19.6		4	17.4		5	21.7	
**Tumor size (pT)**										0.1818[a]
1		21	45.7		12	52.2		9	39.1	
2		19	41.3		10	43.5		9	39.1	
3		4	8.7		1	4.3		3	13.0	
4		2	4.3		0	0.0		2	8.7	
**Nodal status (pN)**										0.1880[a]
0		29	63.0		16	69.6		13	56.5	
1		10	21.7		6	26.1		4	17.4	
2		4	8.7		1	4.3		3	13.0	
3		3	6.5		0	0.0		3	13.0	
**Histology**										0.4599[b]
ductal		32	69.6		15	65.2		17	73.9	
lobular		7	15.2		5	21.7		2	8.7	
others (including mixed types)		7	15.2		3	13.0		4	17.4	
**Molecular subtype**										0.0979[b]
luminal A		24	52.2		15	65.2		9	39.1	
luminal B HER2-		4	8.7		1	4.3		3	13.0	
luminal B HER2+		10	21.7		5	21.7		5	21.7	
nonluminal HER2+		3	6.5		2	8.7		1	4.3	
triple negative/basal-like		5	10.9		0	0.0		5	21.7	
**Stage (AJCC)**										0.0647[a]
I		17	37.0		10	43.5		7	30.4	
II		20	43.5		12	52.2		8	34.8	
III		9	19.6		1	4.3		8	34.8	
**Grade (Nottingham grading system)**										0.2963[a]
1		5	10.9		4	17.4		1	4.3	
2		23	50.0		11	47.8		12	52.2	
3		16	34.8		7	30.4		9	39.1	
unknown		2	4.3		1	4.3		1	4.3	
**ER status**										0.5216[b]
negative		14	30.4		6	26.1		8	34.8	
positive		32	69.6		17	73.9		15	65.2	
**PGR status**										0.0439[b]
negative		12	26.1		3	13.0		9	39.1	
positive		34	73.9		20	87.0		14	60.9	
**HER2 status**										0.7433[b]
negative		33	71.7		16	69.6		17	73.9	
positive		13	28.3		7	30.4		6	26.1	
**Surgery**										0.1528[b]
tumorectomy		10	21.7		7	30.4		3	13.0	
mastectomy		36	78.3		16	69.6		20	87.0	
**Chemotherapy**										0.7425[b]
yes		25	54.3		13	56.5		12	52.2	
no		18	39.1		8	34.8		10	43.5	
unknown		3	6.5		2	8.7		1	4.3	
**Radiotherapy**										0.8329[b]
yes		12	26.1		6	26.1		6	26.1	
no		31	67.4		15	65.2		16	69.6	
unknown		3	6.5		2	8.7		1	4.3	
**Hormonal therapy**										0.3319[b]
yes		33	71.7		18	78.3		15	65.2	
no		10	21.7		3	13.0		7	30.4	
unknown		3	6.5		2	8.7		1	4.3	
**Herceptin**										0.8357[b]
yes		4	8.7		2	8.7		2	8.7	
no		39	84.8		19	82.6		20	87.0	
unknown		3	6.5		2	8.7		1	4.3	

[a)]P-value of Mann-Whitney U test

[b)]P-value of Pearson chi square test

A group of 46 breast cancer patients was divided into two groups: with and without distant metastasis (comparative group) within a follow-up period of 9 years. Baseline demographic and tumor characteristics as well as types of treatment were compared between these two groups using Mann-Whitney U test for continuous (age) and ordinal variables (tumor size, nodal status, stage, grade), and by Pearson chi square test for categorical variables (menopausal status, histology, molecular subtype, ER, PGR and HER2 status, treatment options).

Fig. S1 Dataset legend to Oncomine meta-analysis of *HAX1* expression in breast cancer (ductal and lobular).

1. Invasive Ductal and Invasive Lobular Breast Carcinoma vs. Normal
Curtis Breast, Nature, 2012

2. Invasive Ductal Breast Carcinoma vs. Normal
Curtis Breast, Nature, 2012

3. Invasive Lobular Breast Carcinoma vs. Normal
Curtis Breast, Nature, 2012

4. Ductal Breast Carcinoma vs. Normal
Perou Breast, Nature, 2000

5. Lobular Breast Carcinoma vs. Normal
Perou Breast, Nature, 2000

6. Invasive Ductal Breast Carcinoma vs. Normal
Radvanyi Breast, Proc Natl Acad Sci U S A, 2005

7. Invasive Lobular Breast Carcinoma vs. Normal
Radvanyi Breast, Proc Natl Acad Sci U S A, 2005

8. Ductal Breast Carcinoma vs. Normal
Richardson Breast 2, Cancer Cell, 2006

9. Ductal Breast Carcinoma vs. Normal
Sorlie Breast, Proc Natl Acad Sci U S A, 2001

10. Lobular Breast Carcinoma vs. Normal
Sorlie Breast, Proc Natl Acad Sci U S A, 2001

11. Ductal Breast Carcinoma vs. Normal
Sorlie Breast 2, Proc Natl Acad Sci U S A, 2003

12. Lobular Breast Carcinoma vs. Normal
Sorlie Breast 2, Proc Natl Acad Sci U S A, 2003

13. Invasive Ductal and Lobular Carcinoma vs. Normal
TCGA Breast, No Associated Paper, 2011

14. Invasive Ductal Breast Carcinoma vs. Normal
TCGA Breast, No Associated Paper, 2011

15. Invasive Lobular Breast Carcinoma vs. Normal
TCGA Breast, No Associated Paper, 2011

16. Invasive Ductal Breast Carcinoma vs. Normal
Turashvili Breast, BMC Cancer, 2007

17. Invasive Lobular Breast Carcinoma vs. Normal
Turashvili Breast, BMC Cancer, 2007

18. Invasive Ductal Breast Carcinoma vs. Normal
Zhao Breast, Mol Biol Cell, 2004

19. Lobular Breast Carcinoma vs. Normal
Zhao Breast, Mol Biol Cell, 2004

Fig. S2 *HAX1* overexpression is associated with metastatic relapse (additional data).

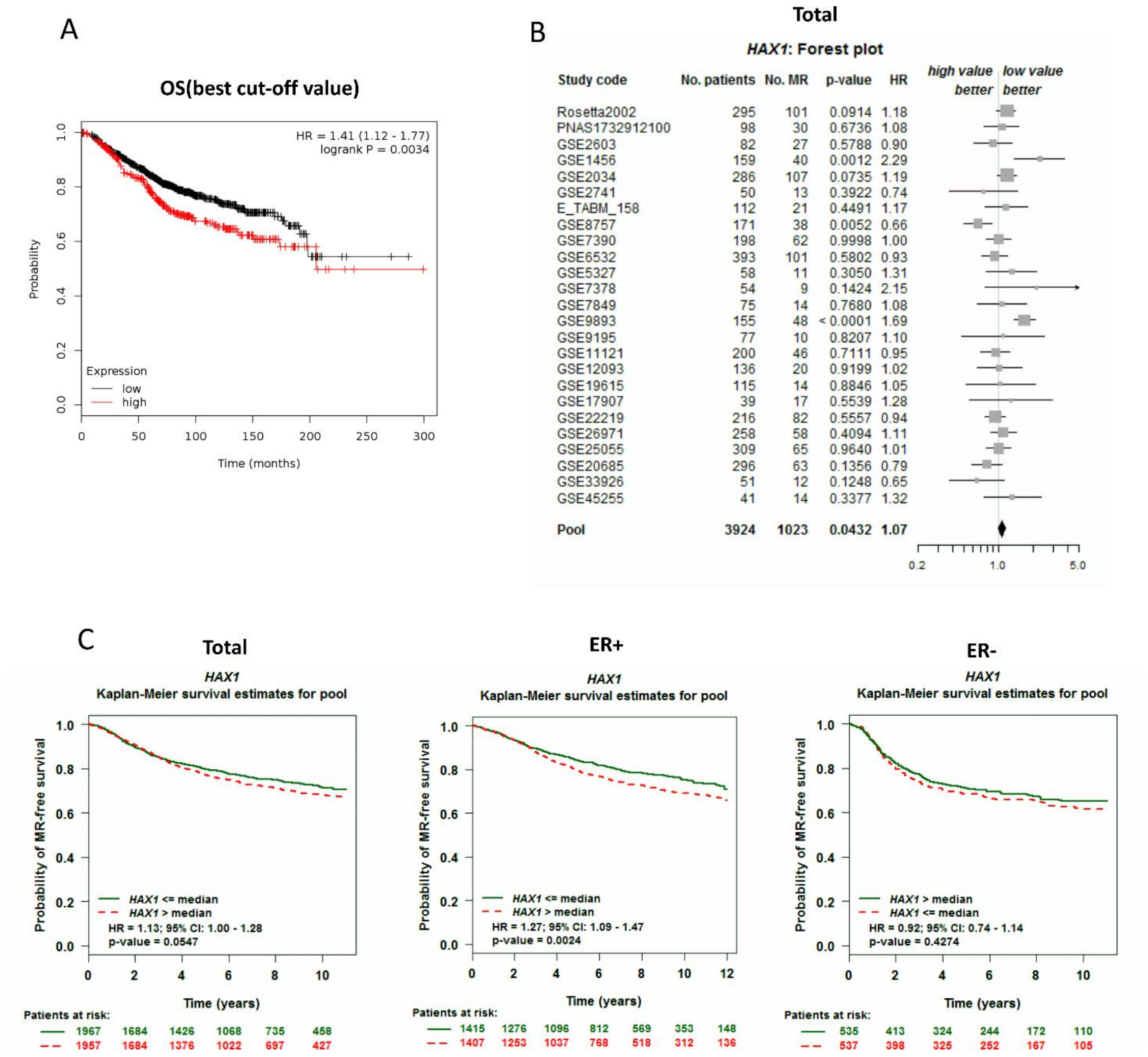

A. Overall survival (OS) analysis for total number of patients (n=1,402). Statistically significant more favorable prognosis for patients with lower *HAX1* expression, but only if patients were split by best cut-off value B. Forest plot for total number of patients with metastatic relapse information (n=3,924) (bcGenExMiner) C. Kaplan-Meier curves for metastatic relapse-free survival (MRFS) plotted in bcGenExMiner for each group of patients with metastatic relapse information (all patients, ER+, ER-). Patients were split by median into groups with high and low *HAX1* expression.
